# Cytomegalovirus microRNAs level determination in kidney recipients post transplantation

**DOI:** 10.1186/s12985-022-01880-5

**Published:** 2022-09-12

**Authors:** Afsoon Afshari, Ramin Yaghobi, Mehdi Golshan

**Affiliations:** 1 Shiraz Nephro-Urology Research Center, Shiraz University of Medical Sciences, Shiraz, Iran; 2 Shiraz Transplant Research Center, Shiraz University of Medical Sciences, Shiraz, Iran

**Keywords:** Cytomegalovirus, Transplant, Kidney, MicroRNA

## Abstract

**Background:**

Human cytomegalovirus (CMV) can establish a latent infection with periodic or sporadic reactivation after the first infection happens. Primary and recurrent infection, results in different problems in patients with impaired or immature immune systems, such as kidney transplant recipients (KTRs). MicroRNAs (miRNAs, miRs) are important regulatory molecules in the outcome of CMV-infected KTRs. Therefore, in this study the expression level of CMV miRNAs were evaluated in active vs. latent CMV infected KTRs.

**Methods:**

Expression of viral miRNAs were studied in 61 KTRs which were divided into 30 active CMV and 31 latent CMV infected individuals. In order to study the expression level of selected miRNAs, SYBR Green Real-time PCR technique was exploited. Also, mature miRNAs expression level that were produced from one precursor, studied both in active and latent situations.

**Results:**

Among studied miRNAs’ expression level, CMV miR-UL112-3p/5p, -UL22A-3p/5p, -US25-1-5p, -US25-2-3p/5p, -UL36-3p/5p and -UL70-3p showed significant increase in active CMV infected KTRs in comparison to latent ones. The ROC curve analysis results for miR-UL112-3p, -UL22A-3p, -US25-2-3p, -UL36-3p and -UL70-3p showed significant difference between two studied patient groups.

**Conclusion:**

This study revealed an extremely high expression level in CMV miR-UL112-3p/5p, -UL22A-3p/5p, -US25-1-5p, -US25-2-3p/5p, -UL36-3p/5p and -UL70-3p in active CMV infected KTRs in comparison to latent ones. Further studies might help in finding the capability of miRNAs to differentiate active from latent stage of CMV infection in KTRs.

**Supplementary Information:**

The online version contains supplementary material available at 10.1186/s12985-022-01880-5.

## Introduction

Human cytomegalovirus (CMV) is a member of the *Betaherpesvirinae* subfamily that discovered in the 1950s [1], which infects up to 60–100% of people in adulthood. This infection might appear after transplantation especially, up to 100 days post kidney transplantation [2]. Kidney transplant recipients (KTRs) have an increased susceptibility to opportunistic infections that might cause a rise in the risk of acute rejection episodes, decrease in graft survival, higher incidence of chronic allograft nephropathy and decrease in the patient survival rate [3, 4]. After detection and studying the regulatory capacities of microRNAs (miRNAs, miRs) of CMV, it seems conceivable that the virus might encode miRNAs for regulating the gene expression pattern in itself and host during productive replication in order to successfully infect, replicate, and stabilize in different cell types [5].

MiRNAs are a class of 19–22 nucleotide noncoding RNAs that have regulatory roles in all classes of metazoans through modulating gene expression during numerous biological processes [6, 7]. CMV is known to have evolved effective immune evasion strategies [8] and CMV-encoded miRNAs have effective immune evasion policies in manipulating the immune responses, which function in interspecies regulation involving viral miRNAs and host genes [9, 10].

The elevation of some miRNA’s expression during latent phase in herpesviruses, produce a nonimmunogenic way for preparing a stable microenvironment suitable for latency [11]. However, expression of CMV miRNAs has been characterized mainly during lytic infection and merely been evaluated during latency either in vivo or in experimental cell culture latency models [12].

The variation in miRNAs' expression pattern is detected in some infectious diseases [13] that turn them to be promising biomarker candidates especially in viral infections. Circulating CMV encoded miRNAs have been considered as diagnostic and discriminative biomarkers in various diseases and their physiological and pathophysiological effects on disease development and progression have been confirmed lately [13, 14]. miR-UL112-3p has been elevated in cardiovascular diseases [14], hypertension and diabetes and glioblastoma multiforme [15], congenital CMV infection [16]. The importance of miR-US25-1-5p as biomarker is discussed in atherosclerosis and congenital CMV infection. Finally, miR-US33-5p have been traced in acute aortic dissection and miR-UL70-3p and miR-US4-5p in glioblastoma multiforme and in hepatitis B, respectively [16].

Currently, CMV infection has been known a serious complication in both hematopoietic stem cell (HSC) and solid organ transplanted (SOT) patients [17]. Some studies are certain about the ability of this CMV miRNA in prediction of replication and/or recurrence of infection specifically in immunocompromised transplanted patients [16].

In view of the CMV-miRNAs role in various situation during viral reactivation in transplanted patients and considering the fact that CMV primary infection and reactivation causes serious problems in KTRs, investigating the CMV-miRNAs expression level in these patients is helpful in handling and understanding the CMV infection outcomes. Therefore, in this study the expression level of CMV miRNAs were evaluated in active vs. latent CMV infected KTRs.

## Materials and methods

### Patients and samples

In this case–control study, 61 kidney transplant patients admitted to Namazi Hospital, Shiraz University of Medical Sciences, Iran, enrolled during 2018–2020. Five ml of EDTA-treated blood samples were collected from each patient in the first week post-transplantations and total PBMCs were extracted from each sample for further analysis. All patients were seropositive for HCMV infection and received anti-HCMV prophylaxis regimen on time transplantation. Patients were divided into two groups based on result of HCMV quantitative Real-time PCR method. Active CMV infected group consists of 30 patients with a positive (more than 10^4^ copy/ml of viral DNA) result and latent group included 31 patients with a negative. This study was approved by the Ethical Committee of Shiraz University of Medical Sciences and also all the used protocols were in conformation with the ethical guidelines of Helsinki Declaration. 30 healthy control samples also were included. The demographical data related to study groups is presented in Additional file 1: Table S1.

All patients were given the same routine regimens of immunosuppressive drugs which consisted of tacrolimus or cyclosporine with mycophenolate mofetil and steroids. The blood level of 200 mg/ml was considered as the therapeutic target for cyclosporin A (CsA; 5 mg/kg/d) or 10 mg/ml of tacrolimus. Donors were selected based on ABO blood group compatibility. HLA matching is performed as a routine procedure for kidney transplanted patients. The including patients were older than 19 years old and all of the patients were tested for other viral infections such as HIV, HSV, HCV, HBV and EBV. The selected samples were negative for mentioned viral infections. Patients showing graft rejecting were excluded from the study. All the samples which composed the active CMV infected group were gathered before starting the anti-viral drugs.

### CMV taq-man real-time PCR

Invisorb Spin Virus DNA Mini Kit (Invitek, Birkenfeld, Germany) was used for CMV-DNA extraction from plasma of all samples according to the manufacturer's instruction. The load of CMV-DNA was determined by using Gensig real-time PCR kit (Primer Design Ltd TM, Advanced kit, United Kingdom). The reaction mix was modified to 20 µl total volume consist of: 500 ng of CMV-DNA, 10 µl of precision TM Master Mix, 1 µl of primers and probe which glycoprotein B (gB) was their target, 1 µl of primers and probe that target the internal control (IC) gene (Applied Biosystems, Grand Island, NY, USA) and finally 3 µl nuclease free water was added. The thermal cycling program used for Step One Plus Real-time PCR thermocycler (Applied Biosystems, Grand Island, NY, USA) was as follow: 95 °C/10 min/1cycle, followed by 95 °C/5 s and then 60 °C/60 s for 50 cycles. This assay was sensitive enough to detect as few as 10 copies of CMV genome/ml of samples.

The copy number of viral DNA was calculated and the patients were divided according the copy number of CMV-DNA. Samples that have more than 10 × 10^3^ copy/ml, were considered to have active viral infection.

### Molecular assays

#### RNA isolation and cDNA synthesis

Total RNA of all samples were extracted from buffy coats using Trizol kit (RiboEx, Korea), with modified protocol. Purity and integrity of extracted RNA was determined by reading 260/280 nm optical density and electrophoresis of RNA on 1% agarose gel.

CDNA synthesis was performed by using a two-step designed procedure. In this procedure universal stem-loop primer (USTLP) is used for cDNA synthesis of all miRNAs. Therefore, in this process in the first step 1 µl dNTP (10 mM), 1 µl USTLP (10 pM), and extracted RNA (100 ng) is mixed in DEPC water until it reaches 10 µl. This mix is heated for 2 min in 65 °C. Then the mix is put on ice for 5 min. The second mix which is composed of 0.5 µl reverse transcriptase enzyme (Takara, Japan) and 2 µl of its 10 × buffer is reached to 10 µl by DEPC water and is added to the first mix. The whole mix is put in 50 °C for one hour for cDNA synthesis accomplished.

#### SYBR green real-time PCR

For Real-time mix SYBR Green Premix (Ex Taq, Takara, Otsa, Shiga, Japan) were used. The mRNA expression level of 12 miRNAs and control gene (U6) was measured using designed primers. The primer sequences, cycling program, and mix contents used for Real-time PCR are presented in Additional file 1: Table S2. Melt curve was analyzed for each reaction to check the specificity of reactions. All data were normalized using the result of control gene amplification (cDNA synthesis and Real-time PCR of miRNAs was done based on the IR patent-101499).

### Statistical analysis

Livak method (2^−ΔΔCt^) was applied to evaluate the expression level of understudy genes in both active and latent CMV infected patients. Statistical analysis was performed by SPSS software ver.24 (SPSS, Chicago, IL, USA) including: non-parametric tests (Mann–Whitney *U* test for studying the *p* value between two study groups, and k independent tests for studying the *p* value between more than two study groups). GraphPad Prism 8 (GraphPad Software, Inc., San Diego, CA, USA) was used to design graphs and calculate the statistical tests. Two-sided Spearman correlation analysis was used to determine the association between miRs arisen from one precursor. Finally, *p* ≤ 0.05 was determined as statistical significance.

## Results

**Fig. 1:**
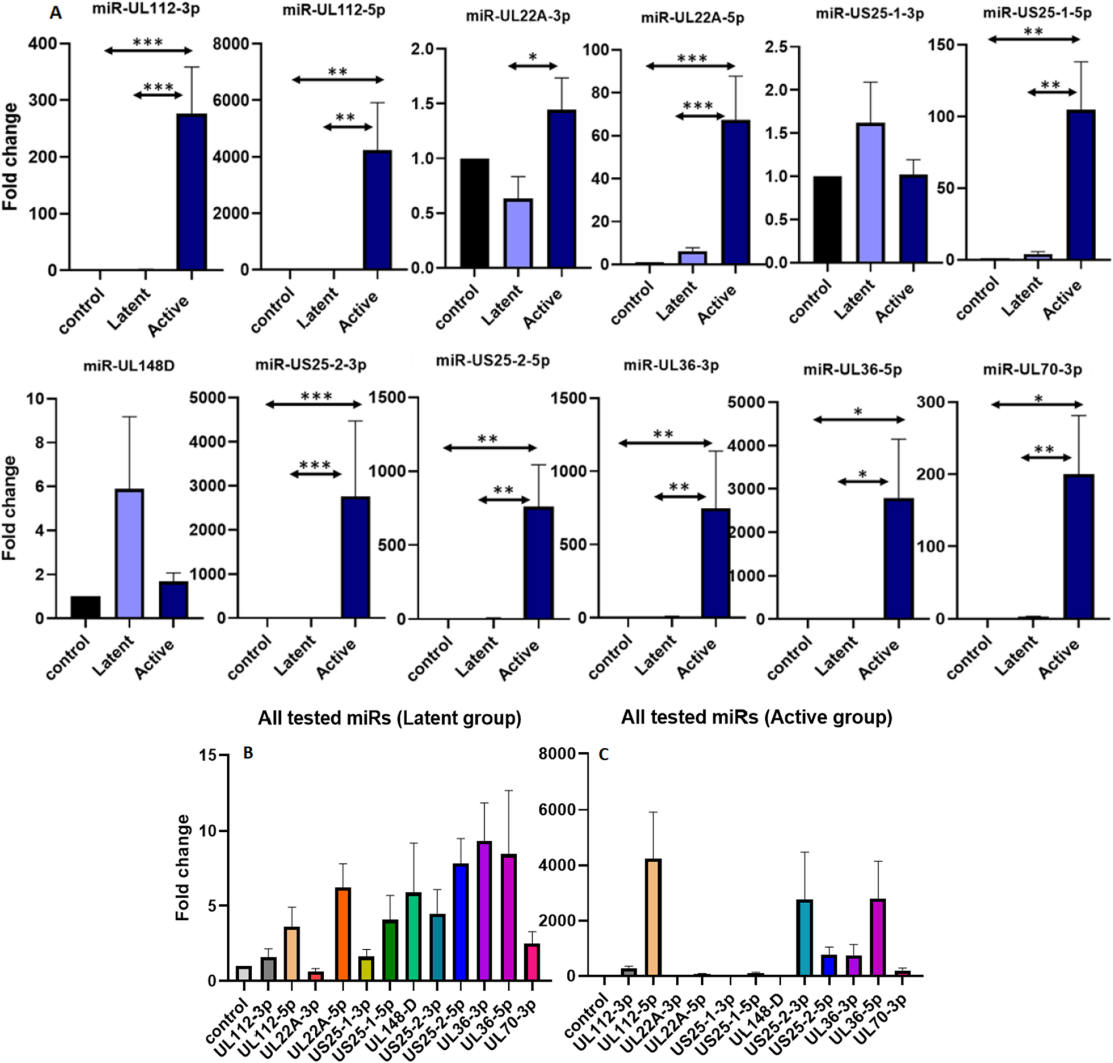
The comparison of miRNAs expression level between latent and active CMV infected groups of patients. **A** Except for miR-US-25-1-3p and miR-UL148D, the expression pattern for all tested miRs were the same and demonstrate increase in expression in the active CMV infected group. Comparison of the expression level of all tested miRNAs in latent CMV infected group (**B**); and Comparison of the expression level of all tested miRNAs in active CMV infected group (**C**); the content of viral miRNAs during activation and inactivation phase of virus life differs completely. *p* < 0.05*, *p* < 0.01**, *p* < 0.001***

The demographic data of studied active versus latent CMV infected groups and controls is presented in Additional file 1: Table S1. Comparing the viral miRNAs gene expression in active versus latent CMV infected patients and control studied groups, showed that most of the CMV miRNAs had a significant increase in active CMV infected KTRs in comparison to latent ones and controls (Fig. 1A). Only miR-US25-1-3p and miR-UL148D had different expression patterns and their maximum expression level is found but not significantly in latent CMV infected group. Furthermore, the expression level of miR-UL22A-3p was decreased in latent CMV infected group in comparison to controls (Fig. 1A). The expression level of all tested miRNAs was compared with each other in latent CMV infected group (Fig. 1B). It is showed that miR-UL36-3p/5p and miR-US25-2-5p has the most expression level in comparison to all other evaluated miRNAs in latent CMV infected patients. The expression level of miRNAs was also compared with each other in active CMV infected group. miR-UL112-5p, miR-US25-2-5p, and miR-UL36-5p has the most expression level in comparison to other miRNAs (Fig. 1C). miR-UL112-3p has several viral and cellular targets. Remarkably, this suggests that single viral miRNAs have the capability to target both cellular and viral gene expression [6] (Additional file 1: Table S5). miR-UL112-3p have the potential of being considered as a differentiating marker between latent CMV infected and active groups of patients.

**Fig. 2:**
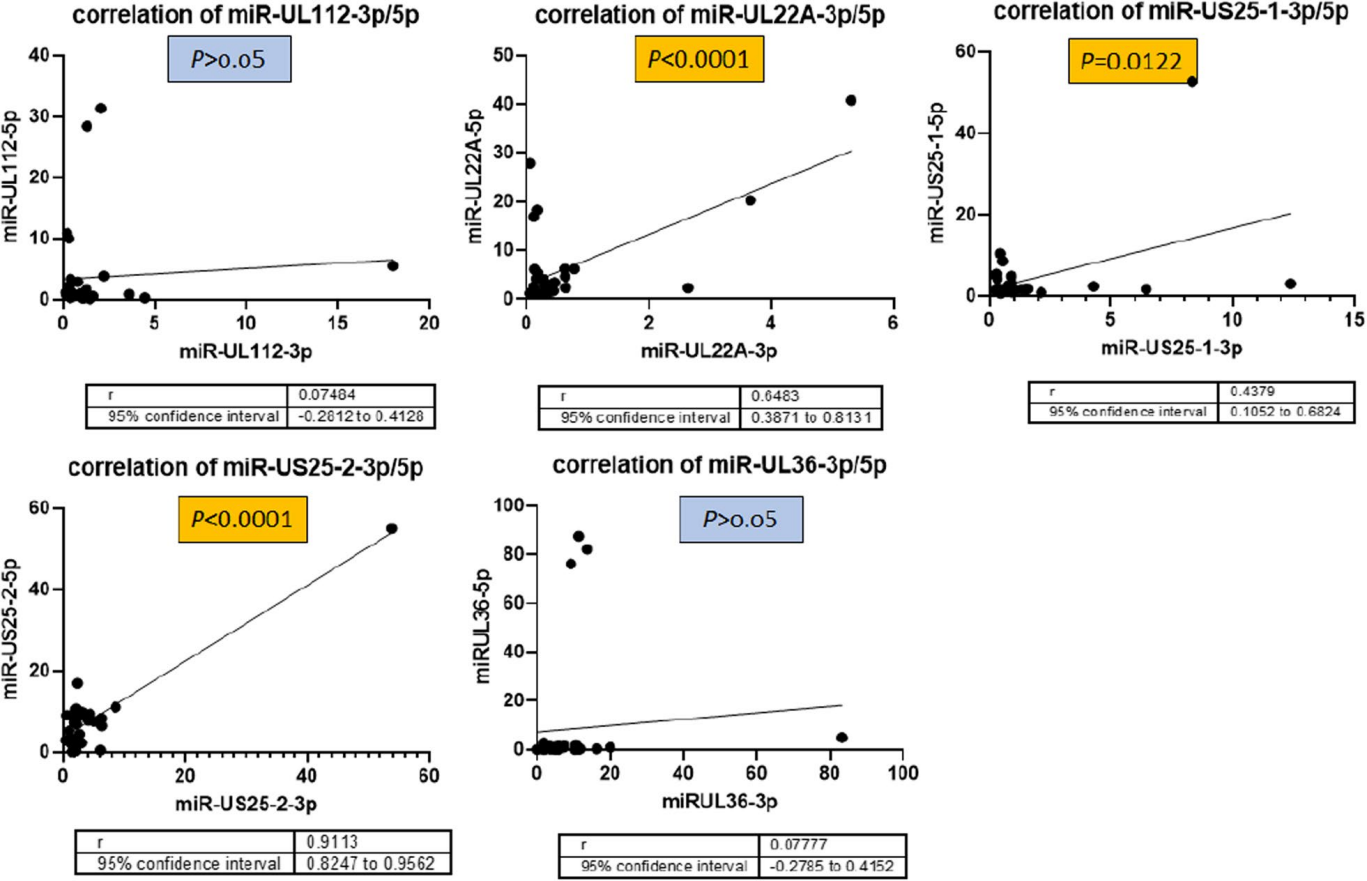
Comparing the correlation between viral precursor miRNAs gene expression in latent CMV infected group, miRNAs that has one precursor arise from the same origin but some of them has different expression rate during inactivation of CMV

Furthermore, the expression level of all tested miRNAs which belong to the same precursor was compared in latent CMV infected group (Fig. 2). Stark et al., found differential incorporation of the 5p and 3p forms of miR-UL112 at 24 hpi (hours post infection) [18]. Correlation analysis of these two miRNAs shows that although these two molecules are derived from one precursor, each can be expressed in different rates due to the infection condition of the target cells. It was previously demonstrated that CMV miRNA precursors incorporate their individual arms into Ago1 and Ago2 differentially [18]. These results show that although miRNAs that has one precursor arise from the same origin but some of them has different expression rate during inactivation of CMV in patients and in this study miR-UL22A-3p/5p, miR-US25-1-3p/5p, and miR-US25-2-3p/5p had significant co-expression pattern in latent CMV infected group.

**Fig. 3:**
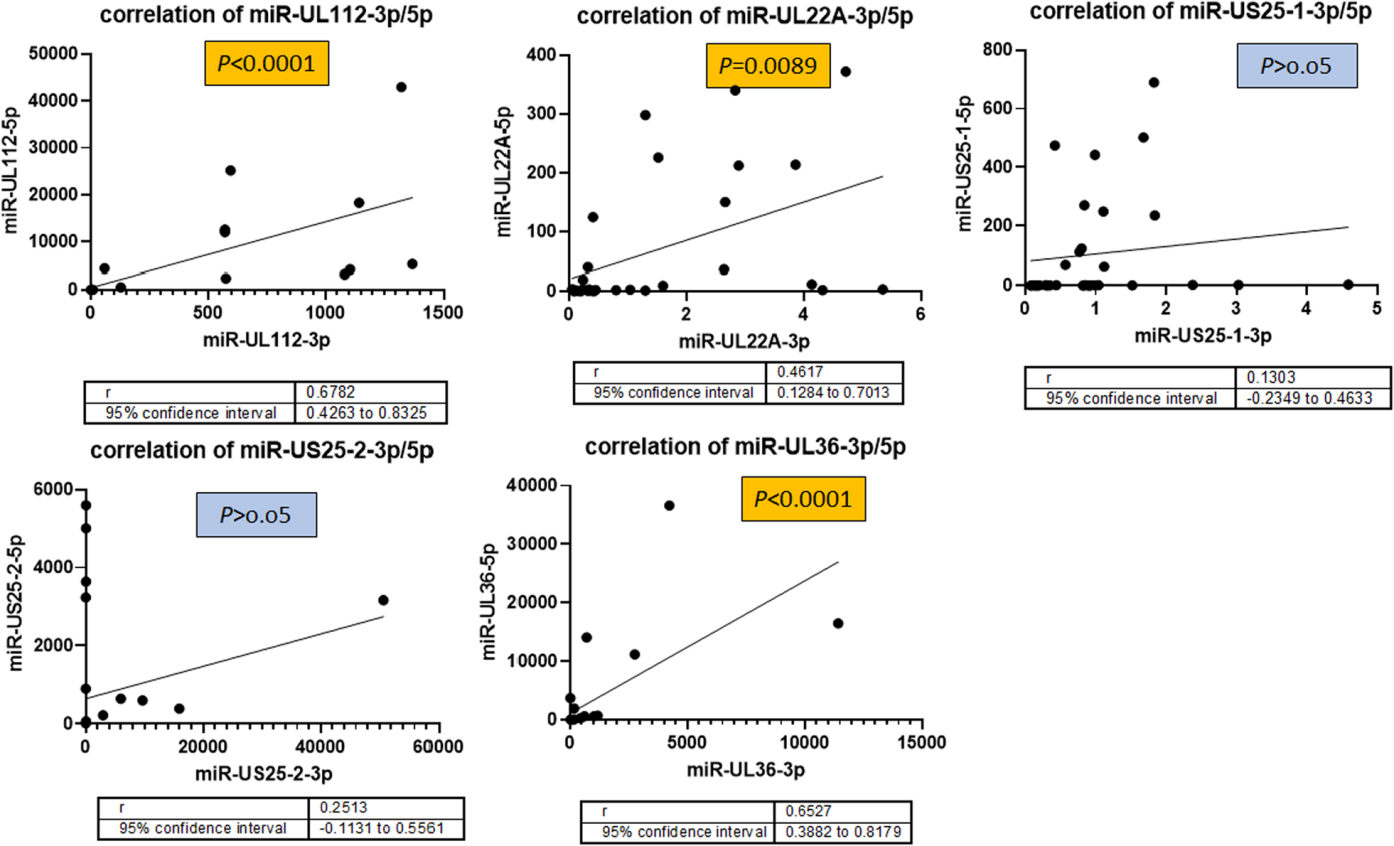
Comparing the correlation between viral precursor miRNAs gene expression in active CMV infected group, miRNAs that has one precursor arise from the same origin but some of them has different expression rate during activation of CMV

Additionally, the expression level of all tested miRNAs which belong to the same precursor was compared in active CMV infected group (Fig. 3). These results show that although microRNAs that has the same precursor, arise from the same origin but some of them has different expression rate during activation of CMV in patients and in the current study, miR-UL112-3p/5p, miR-UL22A-3p/5p, and miR-UL36-3p/5p has significant co-expression pattern in active CMV infected group.

**Fig. 4:**
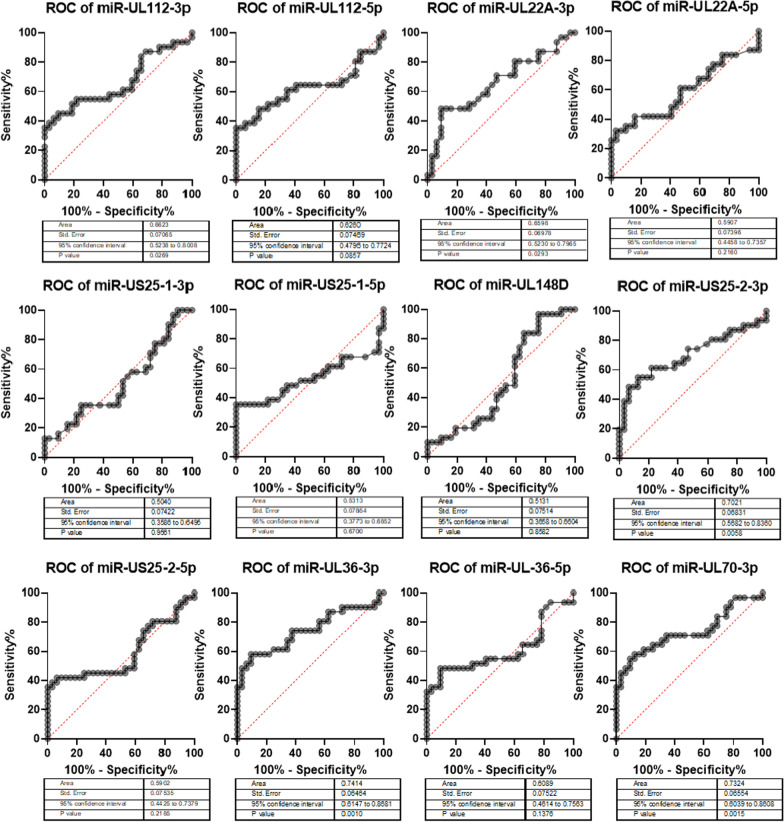
The ROC curve analyses of CMV miRNAs between latent and active CMV infected groups

In order to understand the sensitivity and specificity of each miRNA for differentiating between latent and active CMV infected groups of patients, ROC curve analysis performed. The results in Fig. 4 showed that miR-UL112-3p, miR-UL22A-3p, miR-US25-2-3p, miR-UL36-3p and miR-UL70-3p show significant difference between patient groups (Fig. 4).

The expression level of CMV-miRNAs, were compared to the BUN/Creatinine level of latent CMV infected group and the results showed that only miR-UL36-3p with BUN level and miR-UL112-5p and miR-US25-1-5p with Creatinine level had a statistically significant relation in the patients with latent CMV grade. Additionally, the same study in the active CMV infected group determined that miR-UL112-5p, miR-US25-2-3p and -5p, miR-UL36-3p and -5p and miR-UL70 with the BUN level and all tested miRNAs except miR-UL22A-3p, miR-US25-1-3p, miR-UL148D and miR-UL36-3p had a significant relation with Creatinine level in the patients with active CMV grade. Additionally, the data related to patient condition and BUN/Creatinine level of them are summarized in Tables 1 and 2.

**Table 1:** The detailed condition of selected active CMV infected patients

Number of patients	CMV-DNA viral load (copy number/ml)	Type of transplant	CMV serostatus	Transplant history	BUN (mg/dL)	Creatinine (mg/dL)
1	170,000	Whole	D+/R−	No	30.0	4.00
2	52,000	Whole	D+/R−	No	26.0	3.20
3	138,000	Whole	D+/R−	No	28.5	3.80
4	1,250,000	Whole	D+/R−	No	32.8	4.50
5	800,000	Whole	D+/R−	No	25.0	2.10
6	13,000	Whole	D−/R−	No	21.0	2.70
7	150,000	Whole	D+/R−	No	26.5	3.10
8	45,000	Whole	D+/R−	No	21.8	1.80
9	780,000	Whole	D−/R−	No	19.8	2.60
10	350,000	Whole	D+/R+	Yes	26.5	3.50
11	42,000	Whole	D+/R−	No	15.7	1.40
12	65,000	Whole	D−/R+	No	12.9	1.80
13	660,000	Partial	D+/R+	No	30.8	4.40
14	32,000	Whole	D+/R−	Yes	26.6	3.40
15	14,000	Whole	D+/R−	No	17.1	1.90
16	20,500	Whole	D+/R−	No	18.2	1.50
17	105,000	Whole	D−/R+	No	24.8	2.98
18	120,000	Partial	D−/R+	No	24.8	2.91
19	22,500	Whole	D+/R−	No	19.1	1.60
20	14,500	Whole	D+/R−	No	19.2	1.85
21	17,500	Whole	D+/R−	No	20.8	2.80
22	41,000	Whole	D+/R−	No	14.5	1.90
23	27,000	Whole	D+/R−	No	16.1	2.10
24	55,000	Whole	D+/R−	No	11.5	2.90
25	75,000	Whole	D+/R−	No	10.9	1.10
26	85,000	Whole	D+/R−	No	24.8	1.00
27	20,000,000	Whole	D+/R+	No	22.1	1.90
28	135,000	Partial	D+/R−	No	33.4	4.20
29	230,000	Whole	D+/R−	No	18.5	2.10
30	750,000	Whole	D+/R−	No	12.5	1.70

**Table 2:** The detailed condition of selected latent CMV infected patients

Number of patients	CMV-DNA viral load	Type of transplant	CMV serostatus	Transplant history	BUN (mg/dL)	Creatinine (mg/dL)
1	Negative	Whole	D+/R−	No	56.0	5.10
2	Negative	Whole	D+/R−	No	7.1	2.50
3	Negative	Whole	D+/R−	No	20.9	3.10
4	Negative	Partial	D+/R−	No	31.5	3.80
5	Negative	Whole	D+/R−	No	21.5	4.00
6	Negative	Whole	D−/R−	No	19.8	4.60
7	Negative	Whole	D+/R−	No	27.9	2.40
8	Negative	Whole	D−/R−	No	14.5	1.40
9	Negative	Whole	D−/R−	No	20.9	1.90
10	Negative	Whole	D+/R+	No	23.4	2.80
11	Negative	Whole	D+/R+	No	14.8	1.70
12	Negative	Whole	D−/R−	No	10.9	2.50
13	Negative	Whole	D+/R+	No	28.2	3.70
14	Negative	Whole	D−/R+	No	26.6	2.70
15	Negative	Whole	D+/R−	No	18.9	1.20
16	Negative	Whole	D+/R−	No	17.9	1.80
17	Negative	Whole	D−/R−	No	29.7	2.28
18	Negative	Whole	D−/R−	No	23.1	2.21
19	Negative	Whole	D+/R−	No	18.2	1.90
20	Negative	Whole	D+/R−	No	17.2	1.15
21	Negative	Whole	D+/R+	No	20.8	2.10
22	Negative	Whole	D+/R−	No	14.5	1.20
23	Negative	Whole	D+/R−	No	19.8	1.40
24	Negative	Whole	D−/R−	No	15.9	2.20
25	Negative	Whole	D+/R−	No	11.7	1.40
26	Negative	Whole	D+/R−	No	28.1	1.70
27	Negative	Whole	D+/R+	No	20.9	1.20
28	Negative	Partial	D−/R−	No	25.5	3.50
29	Negative	Whole	D+/R−	No	18.5	1.40
30	Negative	Whole	D+/R−	No	13.8	1.00
31	Negative	Whole	D+/R−	No	56.0	5.10

## Discussion

Earlier, the encoded miRNAs by CMV were detected steadily in host circulating blood. Further studies confirmed the relation between changes in circulating CMV encoded miRNA contents and pathological outcomes in different diseases such as hypertension, cardiovascular disease, cancer, and diabetes, psychiatric and neurological diseases. It is believed that dysregulated viral miRNAs can alter the host transcriptome [19]. The relation between circulating miRNAs of CMV and human diseases was confirmed in essential hypertension for the first time [20]. Reactivation of CMV infection might happen and can cause serious and life-threatening problems in patients harboring impaired or immature immune systems like transplant recipients [21, 22]. miRNAs can promote controlling of viral replication in CMV seropositive SOT recipients' post-transplantation [23]. In current study, the de novo expression of CMV miRNAs during activation and inactivation in KTRs is estimated.

In vitro studies have been confirmed the inhibitory role of CMV miRNAs (US25-1, US25-2-5p, US25-2-3p, and UL112-3p) on the viral replication in the absence of immunosuppression [24, 25]. Therefore, inverse correlations between viral load (DNAmia) and the expression level of miRNAs were expected. Our finding (data not shown) and others [26] could not certify this correlation and proved an association between viral miRNA level and the chance of CMV viremia recurrence after primary treatment, signifies a potential immunoregulatory role for CMV miRNAs. In current study, we found that most of studied viral miRNAs (except for miR-US25-1-3p and UL148D) were increased in the active CMV infected KTRs which is consistent with studies that evaluated the expression of CMV miRNAs in vitro during lytic infection [26]. These results show that the expression level of all studied viral miRNAs except for miR-US25-1-3p and UL148D, have a positive correlation with the increase in the viremia during active CMV infection. Additionally, a study on SOT pre-transplant samples, clarified that expression of some of the host miRNAs (miR-200b-3p and miR-200c-3p) in CMV seropositive patients is correlated to elevation of CMV viremia post-transplantation. The researchers believe that in order to gain the ability of controlling CMV replication post-transplantation, the miRNA responses should be clearly studied [23].

The up-regulation of miR-UL112 that have been confirmed in our study and formerly found in some diseases such as essential hypertension [20], Type 2 diabetes, glioblastoma [15], and SOT patients [26], candidate this CMV miRNA as a novel biomarker for discriminating between CMV latency and active replication in various infected patients [19]. Different targets of miR-UL112-3p (reviewed in Additional file 1: Table S3), miR-UL112-5p (such as caspase 3 and ERAP) [21, 27], miR-UL22A-3p and miR-UL22A-5p (caspase 3 and 7) that are mostly related to production of apoptosis [28].

miR-UL22A induce viral recurrence through down regulation of SMAD3, which is essential in maintenance of latency in infected HPCs (hematopoietic progenitor cells) [29]. Lisboa et al. also, confirmed that miR-UL22A targets C-MYC and results in repression of the heat-shock proteins to support CMV recurrent viremia in SOT patients, propose a new mechanism of immune modulation that help in understanding CMV pathogenesis and might be a candidate biomarker for clinical diagnosis post-transplantation after further evaluations [26].

Our result showed that the expression of miR-UL22A-3p/5p in KT patients upregulates in active CMV infected group. It was interesting that the expression of CMV miR-UL22A-3p in latent CMV infected group was even less than the control group. There are limited studies analyzing the function of CMV miR-UL22A-3p/5p, but it seems that these two miRNAs are managed to establish recurrence of viral infection [30].

CMV miR-US25-1, and miR-US25-2, possibly target cellular genes that might render to viral titer changes [25]. Up-regulation of miR-US25-1-5p, miR-US-25-2-3p, and miR-US-25-2-5p have been verified in SOT patients [26]. Other studies and also ours, indicate that probably miR-US25-1-3p has a slight role in early latent phase expression in order to keep this situation stable, but miR-US25-1-5p might have roles in later expression during activation of viral lytic phase again for reaching a stable latent condition through blocking cell cycle progression by modulating different molecules such as cyclins [27]. CDK6 that is a molecule involved in initiation and maintenance of cell cycle exit during differentiation, and prevents cell proliferation [27] and three groups of genes in association with cell cycle control (including cyclin E1 and E2), as well as histone proteins, and tumor progression are targets of miR-US25-1-3p and -5p, respectively [31].

It was clearly found that over-expression of miR-US25-2-3p results a reduction in CMV replication. miR-US25-2-3p targets eIF4A1 (eukaryotic translation initiation factor 4A1), a vital molecule for cap-dependent translation, cell proliferation and viral growth [24] as well as FAS (a member of apoptosis pathway), CDK6, and CASP3 [27]. By noticing the role of targets of miR-US25-2-3p in reduction of CMV replication and also, by considering the results of our study, it seems that it has a significant role in resuming latency after activation of the virus. Furthermore, the expression rate of CMV miR-US25-2-3p/5p during viral inactivation and activation is different which in latent phase their expression rate is significantly the same while in active duration is not.

Some of the miR-UL148D cellular targets are RANTES (CCL5; that was experimentally confirmed during CMV lytic infection) [32], IEX-1 (suppressed by miR-UL148D and cause inhibition in apoptosis of target cells) [33], IER5 (which can establish latency via increasing CDC25B expression, and limit IE gene expression) [34], ACVR1B (Activin A receptor 1B; is also another target for miR-UL148D during latency and is a part of the activin A signaling cascade) [35]. Previously it was showed that miR-UL148D up-regulates in chronic hepatitis B [36] and Oral lichen planus [37]. Collectively, the data and our results suggest that CMV miR-UL148D function is related to stablish latent situation especially during CMV latent infection rather than lytic [38].

CDK6 and FAS are proved targets for miR-UL36-3p and in this way, it can inhibit apoptosis in target cells [27]. Furthermore, one study presented that miR-UL36-3p has role in promoting efficient lytic replication by down regulating expression of the latency associated protein UL138 and ectopic expression of miR-UL36 enhanced CMV DNA synthesis during the early stages of replication in fibroblasts [39]. SLC25A6 (ANT3) is a western blot proved target for miR-UL36-5p which can cause inhibition in apoptosis pathway [40].

A study found that during early stages of antiviral therapy in SOT patients, increase in the level of miR-UL36-5p is related to the longer persistence of viremia although in in vitro models this miRNA was detected to increase viral replication. The authors also proposed that the mentioned effect might be the result of other opposing forces such as therapeutic immune suppression [15]. Our data also showed that both miR-UL36-3p/5p are significantly increased in active phase of CMV infection.

There are different reports about finding miR-UL70. Stark et al. [18] small RNA-seq data did not support expression of miR-UL70, despite the quantitative PCR experiments by Stern-Ginossar et al. [25] could detect this miRNA. But, Pfeffer et al. [9] also failed to detect this particular miRNA in their cloning efforts. Although some studies were failed in estimating the expression of miR-UL70-3p, Shen et al. [12], evaluated the production of miR-UL70-3p in different cell types and verified the inducing role of this miRNA in CMV replication. In our study we also revealed that the expression of this viral miRNA is significantly increased in active CMV infected KTRs and its importance in maintenance of the viral replication.

Significant correlation was detected only in miR-UL36-3p with BUN level in latent CMV infected KTRs, and miR-UL112-5p, miR-US25-2-3p, miR-US25-2-5p, miR-UL36-3p, miR-UL36-5p and miR-UL70 with the BUN level in active CMV infected KTRs. Only miR-UL112-5p and miR-US25-1-5p had a statistically significant relation with the Cr level in latent CMV infected KTRs. All tested miRNAs except miR-UL22A-3p, miR-US25-1-3p, miR-UL148D and miR-UL36-3p had a significant relation with the Cr level in CMV-activated KTRs.

There were some limitations in our study which it should be into consideration in further studies. We separated buffy coat and plasma of samples but as this was a preliminary report monocytes and dendritic cells weren’t separated and studying these results separately will help us in better understanding the expression pattern of CMV miRNAs during latent and lytic conditions.

## Conclusion

As a matter of fact, the role of miRNAs is detected in both lytic and latent phases of CMV infection. Consequently, in the present study, an extremely high expression level of miR-UL112-3p, -UL112-5p, -UL22A-3p, -UL22A-5p, -US25-1-5p, -US25-2-5p, -UL36-3p, -UL36-5p and -UL70-3p detected in the active CMV infected KTRs in comparison to latent ones, which prove their possible critical and central biological role in the CMV pathogenesis. Finally, it should be considered that the significant changes in viral miRNAs in active vs. latent CMV infected KTRs seems to be of immense importance for further evaluations.

## Supplementary Information

**Additional file 1. Table S1. **The demographic data of studied groups; **Table S2**. The primer sequences, cycling program, and mix contents used for Real-time PCR; **Table S3**. Viral and cellular targets of CMV-miRUL-112-3p.

## Data Availability

The datasets used and/or analyzed during the current study are available from the corresponding author on reasonable request.

## Abbreviations

CMV: Human cytomegalovirus
KTRs: Kidney transplant recipients
miRNAs, miRs: MicroRNAs
NK: Natural killer
MICB: Major histocompatibility complex class I-related chain B
pri-miRs: Primary miRs
eIF4A1: Eukaryotic translation initiation factor 4A1
ACVR1B: Activin A receptor 1B
