# Supplementary material for: Cytomegalovirus microRNAs level determination in kidney recipients post transplantation
Source: Virol J. 2022 Sep 12;19:147. doi: 10.1186/s12985-022-01880-5 (PMC9465962; doi:10.1186/s12985-022-01880-5)
Supplement: Supplementary file 1 — Additional file 1. Table S1. The demographic data of studied groups; Table S2. The primer sequences, cycling program, and mix contents used for Real-time PCR; Table S3. Viral and cellular targets of CMV-miRUL-112-3p. [file 12985_2022_1880_MOESM1_ESM.doc]

**Supplementary Data**

**Journal title: Virology journal**

**Cytomegalovirus microRNAs level determination in Kidney recipients post transplantation**

Afsoon Afshari^1^, Ramin Yaghobi^2*^, Mehdi Golshan^2^

^1^ Shiraz Nephro-Urology Research Center, Shiraz University of Medical Sciences, Shiraz, Iran

^2^ Shiraz Transplant Research Center, Shiraz University of Medical Sciences, Shiraz, Iran

***Correspondence**: Ramin Yaghobi, PhD of Virology,

Shiraz Transplant Research Center, Shiraz University of Medical Sciences, Shiraz; Iran

Tel/Fax: +98-71-3647-3954

E-mail: rayaviro@yahoo.com

**Table S1-** The demographic data of studied groups

|  | | **Latent CMV infected group** | **Activate CMV infected group** | **healthy control**  **group** |
| --- | --- | --- | --- | --- |
| **Underlying diseases** | SLE (Systemic lupus erythematosus) | 7(22.6%) | 5(16.7%) | - |
|  | DKD (diabetic kidney disease) | 11(35.5%) | 11(36.6%) | - |
|  | HTN (hypertension) | 9(29%) | 9(30%) | - |
|  | PKD (Polycystic kidney disease) | 4(12.9%) | 5(16.7%) | - |
| **Gender** | Male | 20 | 18 | 15 |
|  | Female | 11 | 12 | 15 |
| **Age (range; mean)** | Male | 43-68 years; 56.8 | 28-61 years; 43.4 | 25-58 years; 42.7 |
|  | Female | 21-61 years; 39.8 | 25-62 years; 47.5 | 22-51 years; 39.3 |
| **Viral load mean** | - | 0 | 875300 copy/ml | 0 |

**Table S2-** The primer sequences, cycling program, and mix contents used for Real-time PCR

| **Real-time PCR cycling program and mix contents** | **Forward primer sequence (5’→3’)** | **CMV miRNAs ID** | **Number** |
| --- | --- | --- | --- |
| **cycling program:**  95 ^ͦ^c/5min/1cycle followed by 40 cycles of 95 ^ͦ^c/15sec and then 62 ^ͦ^c/35sec  **mix contents:**  10µl of SYBR Green Premix, 0.1µl of ROX dye, forward and reverse primers (each 10pM) and 2µl of synthesized cDNA | CTCGCTTCGGCAGCACA | miR-control | 1 |
|  | AAGTGACGGTGAGATCCAGGC | miR-UL112-3p | 2 |
|  | CCTCCGGATCACATGGTTACT | miR-UL112-5p | 3 |
|  | TCACCAGAATGCTAGTTTGTAG | miR-UL22A-3p | 4 |
|  | TAACTAGCCTTCCCGTGAGA | miR-UL22A-5p | 5 |
|  | TCCGAACGCTAGGTCGGTTCT | miR-US25-1-3p | 6 |
|  | AACCGCTCAGTGGCTCGGACC | miR-US25-1-5p | 7 |
|  | GACATACCGAAGCAACTACCG | miR-UL148-D | 8 |
|  | ATCCACTTGGAGAGCTCCC | miR-US25-2-3p | 9 |
|  | AGCGGTCTGTTCAGGTGGATG | miR-US25-2-5p | 10 |
|  | TTTCCAGGTGTTTTCAACTGT | miR-UL36-3p | 11 |
|  | TCGTTGAAGACACCTGGAAAGA | miR-UL36-5p | 12 |
|  | GGGGATGGGCTGGCG | miR-UL70-3p | 13 |
|  | GTGCAGGGTCCGAGGT | Universal reverse primer | 14 |
|  | GTTGGCTCTGGTGCAGGGT  CCGAGGTATTCGCACCAGAGCCAAC | Stem-loop primer | 15 |

**Table S3-** Viral and cellular targets of CMV-miRUL-112-3p

| **Target gene** | **Viral/cellular**  **target** | **Target function** | **viral latency or lytic** | **Ref.** |
| --- | --- | --- | --- | --- |
| **IE72 (UL123)** | Viral | Downregulates MIE proteins and lowers the kinetic of viral replication. | latency | [1–6] |
| **UL112/113** | Viral | Encodes proteins related to increase viral DNA synthesis. | latency | [7, 8] |
| **UL120 / UL121** | Viral | Downstream of the IE86 ORF and may represent exons within the MIE region. | latency | [9] |
| **UL114** | Viral | Reduction in UL114 protein levels by miR-UL112, reduction in the virus ability to accurately excise uracil residues from viral DNA. | latency | [10, 11] |
| **MICB** | cellular | Cellular ligand for the activating receptor NKG2D which is expressed on NK, γ/δ T, and CD8+ T cells, during cellular stress, such as viral infection, MICB is induced, thus activating NK and T cells result in the killing of infected cells. | latency | [7, 12, 13] |
| **ATG** | cellular | ATG proteins are related to macro-autophagy in the cells that contributes to antiviral defense and restoration of cellular homeostasis during viral infection. | latency | [14, 15] |
| **BclAF1** | cellular | BclAF1 is implicated in apoptosis, transcriptional regulation, RNA processing, and the export of mRNA from the nucleus, BclAF1 level reaccumulates as infection proceeds, but decrease again at late times due to down-regulation by the miR-UL112-3p, reduced BclAF1 enhances CMV gene expression, and its elevated levels inhibit viral replication | latency | [16] |
| **IKKα/β** | cellular | Modulate NF-κB pathway, reduce proinflammatory cytokine secretion | latency | [17] |
| **IL-32** | cellular | reduced IL-32 expression during CMV infection | latency | [18] |

**References:**

1. Sinclair J, Sissons P (2006) Latency and reactivation of human cytomegalovirus. Journal of General Virology 87:1763–1779

2. White EA, Del Rosario CJ, Sanders RL, Spector DH (2007) The IE2 60-Kilodalton and 40-Kilodalton Proteins Are Dispensable for Human Cytomegalovirus Replication but Are Required for Efficient Delayed Early and Late Gene Expression and Production of Infectious Virus. Journal of Virology 81:2573–2583. https://doi.org/10.1128/jvi.02454-06

3. Ahn JH, Hayward GS (1997) The major immediate-early proteins IE1 and IE2 of human cytomegalovirus colocalize with and disrupt PML-associated nuclear bodies at very early times in infected permissive cells. Journal of virology 71:4599–4613. https://doi.org/10.1128/jvi.71.6.4599-4613.1997

4. Korioth F, Maul GG, Plachter B, et al (1996) The nuclear domain 10 (ND10) is disrupted by the human cytomegalovirus gene product IE1. Experimental Cell Research 229:155–158. https://doi.org/10.1006/excr.1996.0353

5. Lukac DM, Harel NY, Tanese N, Alwine JC (1997) TAF-like functions of human cytomegalovirus immediate-early proteins. Journal of virology 71:7227–7239. https://doi.org/10.1128/jvi.71.10.7227-7239.1997

6. Simon CO, Holtappels R, Tervo H-M, et al (2006) CD8 T Cells Control Cytomegalovirus Latency by Epitope-Specific Sensing of Transcriptional Reactivation. Journal of Virology 80:10436–10456. https://doi.org/10.1128/jvi.01248-06

7. Grey F, Nelson J (2008) Identification and function of human cytomegalovirus microRNAs. Journal of Clinical Virology 41:186–191. https://doi.org/10.1016/j.jcv.2007.11.024

8. Park M-Y, Kim Y-E, Seo M-R, et al (2006) Interactions among Four Proteins Encoded by the Human Cytomegalovirus UL112-113 Region Regulate Their Intranuclear Targeting and the Recruitment of UL44 to Prereplication Foci. Journal of Virology 80:2718–2727. https://doi.org/10.1128/jvi.80.6.2718-2727.2006

9. Grey F, Meyers H, White EA, et al (2007) A human cytomegalovirus-encoded microRNA regulates expression of multiple viral genes involved in replication. PLoS Pathogens 3:1593–1602. https://doi.org/10.1371/journal.ppat.0030163

10. Stern-Ginossar N, Saleh N, Goldberg MD, et al (2009) Analysis of Human Cytomegalovirus-Encoded MicroRNA Activity during Infection. Journal of Virology 83:10684–10693. https://doi.org/10.1128/jvi.01292-09

11. Gottwein E, Cullen BR (2008) Viral and Cellular MicroRNAs as Determinants of Viral Pathogenesis and Immunity. Cell Host and Microbe 3:375–387

12. Stern-Ginossar N, Elefant N, Zimmermann A, et al (2007) Host immune system gene targeting by a viral miRNA. Science 317:376–381. https://doi.org/10.1126/science.1140956

13. Nelson JA (2007) Small RNAs and Large DNA Viruses. New England Journal of Medicine 357:2630–2632. https://doi.org/10.1056/nejmcibr0706718

14. Kim S, Seo D, Kim D, et al (2015) Temporal Landscape of MicroRNA-Mediated Host-Virus Crosstalk during Productive Human Cytomegalovirus Infection. Cell Host and Microbe 17:838–851. https://doi.org/10.1016/j.chom.2015.05.014

15. Yordy B, Tal MC, Hayashi K, et al (2013) Autophagy and selective deployment of Atg proteins in antiviral defense. International Immunology 25:1–10

16. Lee SH, Kalejta RF, Kerry J, et al (2012) BclAF1 restriction factor is neutralized by proteasomal degradation and microRNA repression during human cytomegalovirus infection. Proceedings of the National Academy of Sciences of the United States of America 109:9575–9580. https://doi.org/10.1073/pnas.1207496109

17. Hancock MH, Hook LM, Mitchell J, Nelson JA (2017) Human cytomegalovirus microRNAs miR-US5-1 and miR-UL112-3p block proinflammatory cytokine production in response to NF-κB-activating factors through direct downregulation of IKKα and IKKβ. mBio 8:. https://doi.org/10.1128/mBio.00109-17

18. Huang Y, Qi Y, Ma Y, et al (2013) The expression of interleukin-32 is activated by human cytomegalovirus infection and down regulated by hcmv-miR-UL112-1. Virology Journal 10:51. https://doi.org/10.1186/1743-422X-10-51
